# Minimum 8-year follow-up of revision THA with severe femoral bone defects using extensively porous-coated stems and cortical strut allografts

**DOI:** 10.1186/s12891-020-03250-0

**Published:** 2020-04-08

**Authors:** Zi-chuan Ding, Ting-xian Ling, Ming-cheng Yuan, Yong-zhi Qin, Ping Mou, Hao-yang Wang, Zong-ke Zhou

**Affiliations:** 1 Department of Orthopedics, West China Hospital/West China School of Medicine, Sichuan University, 37# Wuhou Guoxue Road, Chengdu, P.R. China; 2 Department of Orthopaedics, The People’s Hospital of Guang’an City, 1# the Fourth Section of Bin He Road, Guang’an, P.R. China

**Keywords:** Revision THA, Femoral bone defects, Extensively porous-coated stems, Cortical strut allografts

## Abstract

**Background:**

Revision total hip arthroplasty (THA) with severe femoral bone defects remains a major challenge. The purpose of this study is to report the minimum 8-year clinical and radiographic results of revision THA with severe femoral bone defects treated with extensively porous-coated stems and cortical strut allografts.

**Methods:**

We retrospectively identified 44 patients diagnosed with Paprosky type III and IV femoral bone defects between January 2006 and July 2011. The exclusion criteria were patients not eligible for surgery, revised with extensively porous-coated stems alone, lost to follow-up and deceased. A total of 31 patients treated with extensively porous-coated stems and cortical strut allografts were finally included in this study. The degree of femoral bone defects was categorized as Paprosky type IIIA in 19 patients, type IIIB in 9 patients and type IV in 3 patients. The mean duration of follow-up was 11.0 ± 1.5 (range, 8.1–13.5) years.

**Results:**

The mean Harris Hip Score improved significantly from 43.4 ± 10.5 points to 85.2 ± 6.6 points (*P* < 0.001). Similarly, WOMAC and SF-12 scores also significantly improved. Twenty-eight stems achieved stable bone ingrowth, two stems showed stable fibrous ingrowth, and one stem was radiologically unstable. Complete union and bridging between cortical strut allografts and host bone was achieved in all 31 patients. The femoral width was augmented with cortical strut allografts after revision surgery (an increase of 10.5 ± 0.5 mm) and showed a slight decrease of 2.5 ± 4.8 mm after the 10-year follow-up. Using re-revision for any reason as an endpoint, the Kaplan-Meier cumulative survival rate of the stem was 96.2% (95% confidence interval, 75.7–99.5%) at 10 years.

**Conclusion:**

Our data demonstrate that the use of extensively porous-coated stems combined with cortical strut allografts in revision THA with Paprosky type III and IV femoral bone defects can provide satisfactory clinical and radiographic outcomes with a minimum follow-up of 8 years.

## Background

Femoral bone defects that must be addressed at the time of revision total hip arthroplasty (THA) may result from aseptic loosening, infection, osteolysis, periprosthetic fracture, stress shielding and implant removal [1]. The primary goal of femoral revision is to obtain initial stability of the stems with the ultimate objective being long-term implant survivorship and the restoration of hip function. Paprosky type III and IV femoral bone defects exhibit severe proximal metaphyseal bone defects and varying extents of diaphyseal bone defects. The odds of achieving stable proximal fixation in the presence of severe metaphyseal bone defects are unreliable. Furthermore, the remaining diaphyseal bone may be inadequate to support the components, and achieving distal fixation may be difficult, making the revision more challenging. In addition, in revision THA, poor femoral bone stock influences functional outcomes, increases the risk of mechanical failure and periprosthetic fracture, and is associated with particular problems if further revision is required [2–5]. The reliable long-term durability of revision components and the restoration of hip function are of vital importance in revision THA, since the number of relatively young patients with a long-life expectancy increases.

Cementless extensively porous-coated stems can bypass the proximal femoral bone defect region and achieve scratch fit fixation depending on 5–7 cm of the diaphysis and have produced reliable clinical and radiographic results in revision THA with femoral bone loss [6]. However, the application of extensively porous-coated stems in femurs with type III and IV defects remains a concern because the bone defects extend to the diaphysis, and the residual diaphyseal bone may be inadequate for distal fixation [7, 8]. In such cases, extensively porous-coated stems, when used alone, may lead to a high rate of failure, and other alternative methods are required to provide stable fixation [7, 8]. Cortical strut allografts can provide secure initial stability for extensively porous-coated stems and further restore femoral bone stock [9–11]. A combination of extensively porous-coated stems and cortical strut allografts is an effective way to reconstruct a femur with severe bone defects. Satisfactory short-term outcomes (after a mean follow-up of 2–5 years), including high survivorship of stems, a high rate of allograft incorporation and the successful restoration of bone stock, have been reported for this technique [9–12]. However, potential concerns regarding the durability of extensively porous-coated stems with fibrous fixation and resorption of cortical strut allografts over longer follow-up periods have been raised.

The purpose of this study is to report the mean 10-year clinical and radiographic results of revision THA with Paprosky type III and type IV femoral bone defects treated using extensively porous-coated stems and cortical strut allografts.

## Methods

### Patients

This retrospective study was approved by the institutional review board of our hospital, and informed consent for participation was obtained from all patients. We identified patients from the departmental database that prospectively collected patient information. The inclusion criteria were patients who underwent revision THA with Paprosky type III and IV femoral bone defects using extensively porous-coated stems and cortical strut allografts between January 2006 and July 2011. Patients revised with extensively porous-coated stems alone were excluded. Patients not eligible for surgery or who were lost to follow-up or deceased were also excluded. From January 2006 to July 2011, a total of 44 patients with a diagnosis of Paprosky type III and IV femoral bone defects were treated at our institution. One 85-year-old female patient with poor general health was assessed as ineligible for surgery and excluded from this study. Four patients were managed with extensively porous-coated stems alone and excluded. A total of 39 patients with extensively porous-coated stems and cortical strut allografts were identified. Five of the 39 patients were lost to follow-up at the latest follow-up, and two of the 39 patients were deceased (no deaths were related to the revision surgery). One patient was reached by telephone at the last follow-up, and he was satisfied with his hip function but refused to return for follow-up. The remaining 31 patients (all with unilateral revision THA) were included in this study.

The demographic data of the patients are summarized in Table 1. The most common reason for revision was periprosthetic femoral fracture (PFF), followed by aseptic loosening (AL) and periprosthetic joint infection (PJI). Of the 12 PFF patients, 8 were caused by falling to the floor, 2 were caused by an accident, and the others occurred spontaneously. PFFs were all classified as Vancouver-type B3. The degree of femoral bone defects was categorized according to the criteria of Paprosky et al. [13] Among the 31 patients, 19 were categorized as Paprosky type IIIA, 9 as type IIIB and 3 as type IV. The mean duration of the follow-up was 11.0 ± 1.5 (range, 8.1–13.5) years.

**Table 1:** Demographic data

Parameters	Numbers
No. of patients	31
Male: female (no. of pts)	19: 12
Age (years) ^a^	62 (32)
Primary diagnosis (no. of pts., %)	
Osteonecrosis of the femoral head	16 (51.6%)
Developmental dysplasia of the hip	7 (22.6%)
Primary osteoarthritis	5 (16.1%)
Femoral neck fractures	2 (6.5%)
Rheumatoid arthritis	1 (3.2%)
Time from primary to revision THA (years) ^a^	6.4 (7.8)
Reason for revision (no. of pts., %)	
PFF	12 (38.7%)
AL	10 (32.3%)
PJI	9 (29.0%)
Fixation of previous femoral stems (no. of pts., %)	
Cement	20 (64.5%)
Cementless	11 (35.5%)
Degree of femoral bone defects (Paprosky classification)	
Type IIIA	19 (61.3%)
Type IIIB	9 (29.0%)
Type IV	3 (9.7%)
ETO utilized	7 (22.6%)
Follow-up (years) ^b^	11.0 ± 1.5 (range, 8.1–13.5)

*AL* Aseptic loosening, *PJI* Periprosthetic joint infection, *PFF* Periprosthetic femoral fracture, *ETO* Extended trochanteric osteotomy

^a^ Skewed distribution data are presented as medians with interquartile ranges

^b^ Normal distribution data are presented as the mean ± standard deviation

### Treatment method

Patients undergoing revision THA complained of unbearable hip pain and unacceptable hip function before revision surgery. Through a preoperative evaluation including a comprehensive history, physical examination, laboratory tests and radiographs, the preoperative diagnosis was made. PFF, AL and PJI were the most common indications for femoral component revision at our institution. Then a revision THA was contemplated and performed. All femoral component revisions were performed using extensively porous-coated stems with or without cortical strut allografts at our institution. The decision to use cortical strut allografts was made intraoperatively when the initial axial and rotational stability of the extensively porous-coated stems could not be achieved because of severe bone defects. If the host bone can provide reliable initial stability for the extensively porous-coated stems, the stems were used alone. No other techniques, such as tapered stem, allografts with prosthetic composites (APC) or proximal femoral replacement, were ever used at our institution.

### Surgical technique

Before surgery, anteroposterior and lateral radiographs of the femur were utilized to assess the stability of the stems and femoral bone stock. If necessary, CT scans were taken to further evaluate the severity of femoral bone defects. A posterolateral approach was used for all patients. An extended approach was used to expose the femoral bone defect sites or fracture sites when necessary. The stability of the previous acetabular cup was evaluated intraoperatively. A total of 27 patients with loose cups underwent acetabular component revision. All femoral components were revised with cementless extensively porous-coated Solution stems (DePuy, Warsaw Indiana), which are monoblock seven-eighths porous-coated stems with a cylindrical distal end.

Extended trochanteric osteotomies (ETOs) were performed in seven patients to remove the previous stems. Then, reamers that gradually increased in size were used to prepare the femoral canal until the diaphyseal cortex was involved. After the trial stems were inserted, their axial and rotational stability were assessed by applying traction force and rotation force, respectively. The initial axial and rotational stability of the trial stems could not be achieved without cortical strut allografts because of poor bone quantity and quality in all 31 patients. As a result, we decided to use cortical strut allografts to provide additional stability for the stems. Two or three strut allografts with a mean length of 16.1 cm were shaped to fit closely against the surface of the patient’s femur and placed in different planes distally. After the strut grafts were completely tightened by double-loop cerclage wires, the real stems were inserted. All stems achieved definitive intraoperative initial stability after the use of cortical strut allografts. Bridging cortical bone loss areas and supporting thinning cortex with strut allografts before inserting the stems may also reduce the risk of intraoperative fracture. With regard for PFF patients, applying strut allografts before insertion of the stems also strengthened the fixation of the fracture and prevented re-fracture. Cortical strut allografts were appropriately placed to ensure that the fracture lines were exceeded by over 5 cm distally. Bone defects in the medullary cavity and the gap between cortical strut allografts and host bone were filled with cancellous allografts. The allografts used in this study had been stored at − 80 °C for at least 3 months in the bone bank of our institution. The allografts were repeatedly soaked in povidone-iodine solution and finally coated with dry powdered gentamicin and vancomycin; all of these procedures were performed on another surgical table under sterile conditions.

All patients were encouraged to conduct isometric exercises and active motions while in bed immediately after surgery. All patients were treated with antibiotic prophylaxis and deep venous thromboembolism prophylaxis postoperatively [14]. Generally, the patients were mobilized with partial weight-bearing at 1 to 4 weeks after surgery depending on the degree of bone defect. Full weight-bearing and ambulation without crutches were allowed after 4 to 12 weeks. Patients were followed up regularly at our institution after surgery.

### Clinical assessment

At the latest follow-up, clinical evaluation was conducted by two observers using the Harris Hip Score (HHS), the Western Ontario and McMaster Universities Osteoarthritis Index (WOMAC, covering pain, stiffness and function scores) and the SF-12 scale (covering physical component summary and mental component summary). The total WOMAC scores, WOMAC pain scores, WOMAC stiffness scores, WOMAC function scores, SF-12 PCS scores and SF-12 MCS scores were normalized to a range of 0 to 100 points, with higher scores indicating better function. Any complications during or after surgery were recorded.

### Radiographic assessment

Anteroposterior and lateral radiographs of the femurs, including full-length stems, were taken and reviewed at each follow-up time point. The fixation and stability of the cementless femoral component was evaluated according to the criteria of Engh et al. [15] Subsidence of the stem was measured as previously described [16]. Radiolucent lines around the stems were divided into seven zones as described by Gruen et al. [17] Nonunion of a PFF and ETO were defined as a persistent fracture line or the absence of a bridging callus, respectively, at 6 months postoperative [18]. The incorporation of cortical strut allografts into host bone was defined as complete union and bridging between them. Resorption of strut allografts was graded as follows according to Maury et al.: mild when the partial-thickness resorption of one cortex was less than 1 cm in length, moderate when the partial-thickness resorption of one cortex was more than 1 cm in length, and severe when full-thickness resorption of the cortex was observed [19]. Femoral width was measured at the zone with the most severe bone loss observed on anteroposterior radiographs, where the strut allografts were always applied to augment the bone stock [20]. To minimize potential errors in the measurement of femoral width caused by femoral rotation, all preoperative and postoperative radiographs were taken using a standard protocol. The patients were positioned in an anatomically supine position. Their feet were placed together with the ankle at 15° of internal rotation and the patella facing the ceiling. The x-ray tube was placed over the patients 100 cm from and perpendicular to the table. In addition, the same series of radiographs was carefully examined for femoral rotation by comparing the profile of the less trochanter and prothesis.

### Statistical analysis

Demographic data and outcomes were evaluated for a normal distribution using histograms and the Kolmogorov-Smirnov test prior to analyses. Continuous variables with a normal distribution are presented as the mean ± standard deviation and were analyzed with t tests. Continuous variables with skewed distributions are presented as medians with interquartile ranges (IQRs) and were analyzed with the Mann–Whitney U test. Categorical variables are presented as absolute values (percentages) and were analyzed with the Chi-square test. The level of significance was defined as *p* < 0.05. Kaplan-Meier survivorship analysis was performed to analyze the cumulative survival rate of the stem. The end point for survival was defined as re-revision for any reason. Statistical analysis was performed with SPSS v22.0 (IBM, Armonk, NY).

## Results

### Clinical outcomes

With regard for hip function and quality of life at the most recent follow-up, all patients showed significant improvement after revision THA. (Table 2) The mean Harris Hip Score improved significantly from 43.4 points to 85.2 points (*P* < 0.001). Similarly, WOMAC and SF-12 scores also significantly improved. One patient (3.2%) needed a re-revision surgery for AL 9 years after the index revision surgery. The failure rates were 0% (0 out of 7) in patients with ETO and 4.2% (1 out of 24) in patients without ETO, and there was no significant difference between the groups (*P* = 0.575). Thigh pain was observed in only three patients (9.7%).

**Table 2:** Clinical results

Parameters	Preoperative	Postoperative	*P* Value
Harris hip score			
Mean in points^a^	43.4 ± 10.5	85.2 ± 6.6	< 0.001
Rating			< 0.001
Excellent (90–100 points)	0 (0%)	17 (54.8%)	
Good (80–89 points)	0 (0%)	11 (35.5%)	
Fair (70–79 points)	1 (3.2%)	3 (9.7%)	
Poor (< 70 points)	30 (96.8%)	0 (0%)	
WOMAC^a^			
Total	39.1 ± 17.3	75.9 ± 10.4	< 0.001
Pain	41.3 ± 18.2	82.0 ± 10.8	< 0.001
Stiffness	50.5 ± 23.4	76.9 ± 16.4	0.002
Function	36.9 ± 17.2	75.1 ± 12.9	< 0.001
SF-12^a^			
PCS	30.9 ± 14.7	55.9 ± 18.3	< 0.001
MCS	34.2 ± 13.7	60.0 ± 21.9	< 0.001
Walking without aids	0 (0%)	23 (74.2%)	< 0.001
Thigh pain	–	3 (9.7%)	
Satisfaction			
Very satisfied	–	23 (74.2%)	
Satisfied	–	5 (16.1%)	
Neutral	–	2 (6.4%)	
Dissatisfied	–	1 (3.2%)	
Very dissatisfied	–	0 (0%)	

*PCS* Physical component summary, *MCS* Mental component summary; The total WOMAC scores, WOMAC pain scores, WOMAC stiffness scores, WOMAC function scores, SF-12 PCS scores and SF-12 MCS scores were normalized to a range of 0 to 100 points, with higher scores presenting better function

^a^ Normal distribution data are presented as the mean ± standard deviation and were analyzed with t test

Intraoperative fractures occurred in two patients who were treated with internal fixation using cerclage wires. The fractures united successfully after surgery with no need for a further procedure, and no sign of stem loosening or re-fracture was observed at the latest follow-up. One superficial wound infection was observed and treated with antibiotics. The wound healed successfully with no sign of deep infection, and no further wound complications occurred in the patient. One patient experienced postoperative dislocation 3 days after surgery and was successfully treated with closed reduction. No recurrence of dislocation was observed at the latest follow-up. No further complications occurred, and no re-revision was required in these patients. No PJI occurred after revision surgery.

### Radiographic evaluation

**Fig. 1:**
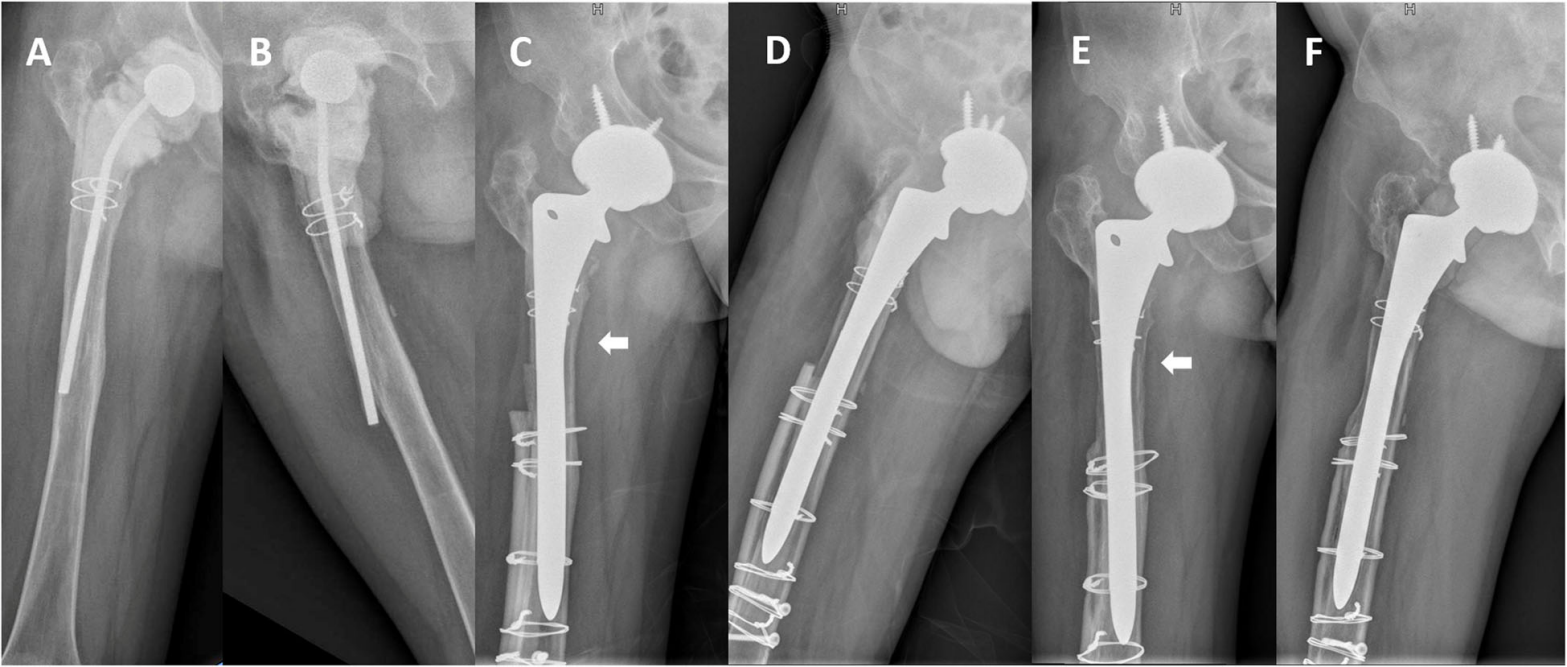
**a-b** Preoperative radiographs of a 38-year-old man who underwent revision THA for periprosthetic joint infection, showing the placement of antibiotic-loaded spacer with Paprosky type IIIA femoral bone defect. **c-d** Radiograph immediately after revision with extensively porous-coated stem and cortical strut allografts. **e-f** Radiographs at 9 years after revision. Bone ingrowth and no signs of stem loosening were observed. Cortical strut allografts incorporated to the host bone successfully. Moderate stress shielding was observed in proximal femur both medial and lateral sides (white arrow)

Among the 31 stems, 28 achieved stable bone ingrowth, two showed stable fibrous ingrowth, and one was radiologically unstable. (Table 3) (Fig. 1) The two stems with fibrous ingrowth showed extensive radiolucent lines in Gruen Zone 1 and 7, severe bone stock deficiency and no obvious endosteal spot weld around the porous surface. However, no signs of stem subsidence were found at the latest follow-up. As a result, we assessed these two stems as stable fibrous ingrowth. An implant that showed a subsidence of 30 mm and extensive radiolucent lines in Gruen Zone 1, 2, 6 and 7 was assessed as unstable. All stems showed subsidence less than 5 mm except for the loosening stems.

**Table 3:** Radiographic results

Parameters	Numbers
Fixation and stability of stems (no. of pts., %)	
Stable bone ingrowth	28 (90.3%)
Stable fibrous ingrowth	2 (6.4%)
Unstable	1 (3.2%)
Incorporation of cortical strut (no. of pts., %)	31 (100%)
Resorption of cortical strut (no. of pts., %)	
Mild	23 (74.2%)
Moderate	8 (25.8%)
Severe	0 (0%)
Femoral width (millimeters)^a^	
Pre-op	31.7 (8.9)
IM po-op	42.4 (12.1)
FU	38.9 (8.2)
Changes in femoral width (P Value)**	
IM po-op vs Pre-op^a^	10.5 ± 0.5 (p < 0.001)
FU vs Pre-op^a^	7.8 ± 5.6 (p < 0.001)
IM po-op vs FU^a^	2.5 ± 4.8 (*p* = 0.002)

*Pre-op* Preoperative, *IM po-op* Immediate postoperative, *FU* Ten years of follow-up

^a^Skewed distribution data are presented as medians with interquartile ranges

**The changes in femoral width were calculated by subtracting the latter from the former. The data were normally distributed and are presented as the mean ± standard deviation. *P* values were analyzed with t tests

**Fig. 2:**
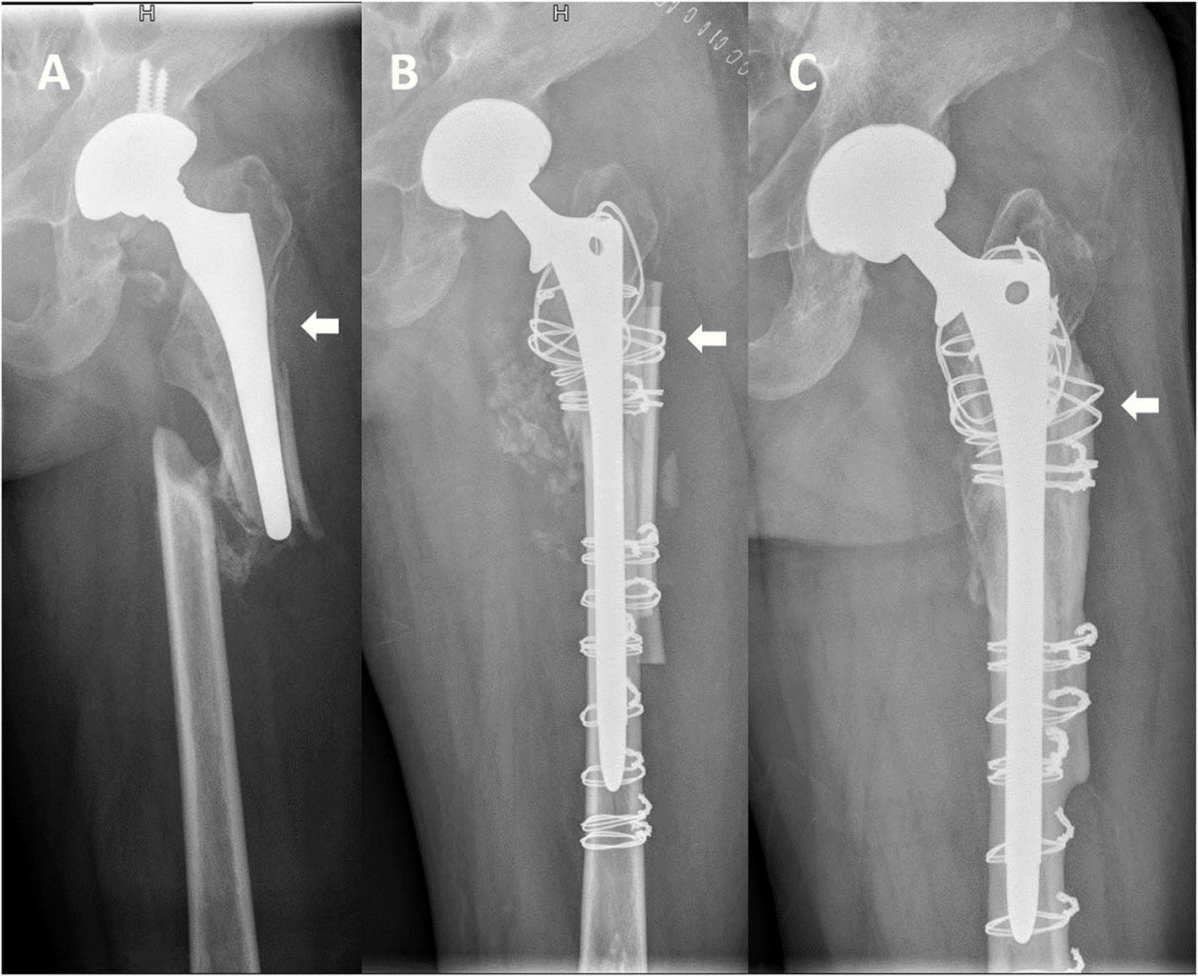
**a** Preoperative radiographs of a Vancouver-type B3 periprosthetic femoral fracture. **b** Radiograph at postoperative day 1. Periprosthetic femoral fracture was treated with extensively porous-coated stem and cortical strut allografts. **c** At 10-year follow-up, no subsidence and radiolucent lines was identified and the stem was considered to be bone ingrowth stable. Bone restoration of bone defect area (white arrow) was observed. Cortical strut allografts incorporated to the host bone successfully and the resorption of cortical strut was assessed as mild

Among all 31 patients, complete union and bridging between the cortical strut allografts and host bone were achieved, suggesting the successful incorporation of cortical strut allografts into host bone. (Fig. 2) Resorption of the cortical strut allografts was graded as mild in 23 patients and moderate in 8 patients, and no strut allografts were assessed as exhibiting severe resorption. The femoral width was increased by a large margin by the cortical strut allografts after revision surgery and had slight decreased after 10 years of follow-up. (Table 3) All PFFs and ETOs achieved union.

### Survivorship analysis

**Fig. 3:**
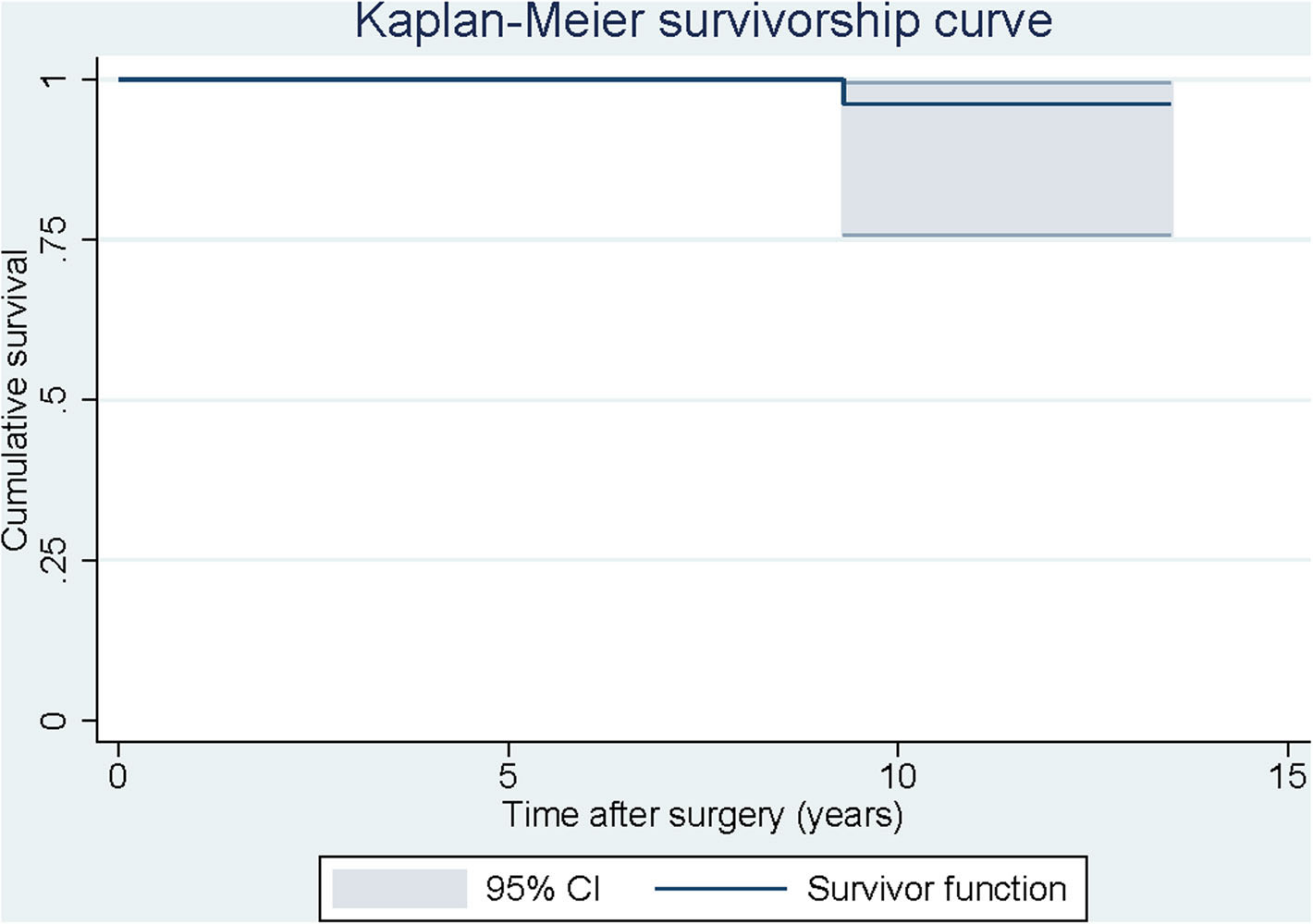
Kaplan-Meier survivorship curve with re-revision for any reason or radiographic signs of loosening as end points

During the follow-up period, one patient needed a re-revision surgery for AL at 9 years after the index revision surgery. Using re-revision for any reason as an end point, the Kaplan-Meier cumulative survival rate at 10 years was 96.2% (95% confidence interval, 75.7–99.5%). (Fig. 3).

## Discussion

Performing femoral component revision in Paprosky type III and IV bone defects continues to be a major challenge. The most important finding of our study is that combining extensively porous-coated stems with cortical strut allografts provided satisfactory clinical and radiographic outcomes for patients undergoing revision THA with Paprosky type III and IV femoral bone defects after a minimum follow-up of 8 years. Stems with fibrous fixation could be durable when supported by cortical strut allografts, and the survival rate of the stems at 10 years was 96.2%. All the cortical strut allografts achieved union and the significant improvement in bone stock was stable after a minimum follow-up of 8 years.

Obtaining and maintaining the stability of the revision femoral component in the presence of such bone defects are difficult. Various techniques are available for the management of revision THA with severe bone defects, and these include cemented stems, cementless proximally porous-coated stems, extensively porous-coated stems, tapered stems, APC and proximal femoral replacement. Cemented stems and cementless proximally porous-coated stems did not achieve satisfactory outcomes in revision THA with severe bone loss since they depend on the proximal metaphyseal for fixation [1]. Modular and monoblock tapered stems have been reported to have excellent results in revision THA with Paprosky type III and IV defects [21]. Modular tapered stems can equalize limb lengths and optimize offset and anteversion and are correspondingly thought to have better clinical outcomes. However, concern persists regarding the risk of mechanical failure in the application of tapered stems, especially failure of the junction in modular design [22, 23]. The use of APC has been reported to contribute to satisfactory long-term results for severe proximal bone defect management in revision THA [24]. Nevertheless, the difficulty of obtaining allografts, the requirement for higher techniques and the risk of nonunion and spreading disease limit the extensive use of APC [25]. Proximal femoral replacement with a megaprosthesis is another way to obtain initial stability in revision THA with severe femoral bone defects. However, proximal femoral replacement has disadvantages, including postoperative dislocation, a high risk of aseptic loosening and further bone loss [26].

Paprosky et al. retrospectively reviewed 170 patients who underwent revision THA with bone defects using extensively porous-coated stems [6]. After a mean follow-up of 13.2 years, only 7 mechanical failures had occurred, and the survival rate of the stem at 16 years was greater than 95%. Recent studies have reported similar low re-revision rates for extensively porous-coated stems in revision THA that range from 0 to 15% after a mean follow-up of 2.1 to 16.1 years [6, 9–12, 27–32]. (Table 4) However, the application of extensively porous-coated stems alone in revision THA with type III and IV defects remains a concern because the residual diaphyseal bone may be unable to provide secure stability for the stems [7, 8]. Cortical strut allografts can support and provide initial stability for extensively porous-coated stems and can also be used for the treatment of bone deficiency in revision THA [9, 11, 32]. While the short-term results of extensively porous-coated stems combined with cortical strut allografts in revision THA with femoral bone defects have been encouraging, [9–11] few studies have reported longer follow-up results. Kim et al. reported that in 120 patients with severe femoral bone defects, when combined with cortical strut allografts, the survival of extensively porous-coated stems was 91% at 16 years of follow-up [32]. In the current study, we reported similar excellent outcomes for this technique in the management of revision THA with Paprosky type III and IV femoral bone defects at a mean follow-up of 10 years.

**Table 4:** Cementless extensively porous-coated stem with or without cortical strut allografts in revision THA in the literature

Study	Year	Type of cementless extensively porous-coated stem	Cortical strut allografts usage	Patients (hips)	Mean follow-up (years)	Paprosky classification of bone defects	Incorporation of cortical strut allografts	Clinical results	Radiographic results	Re-revision	Survival
Paprosky et al.	1999	Monoblock: AML and Solution	NA	170	13.2 (range, 10–16)	Type I: 18 Type II: 51 Type IIIa: 82 Type IIIb: 19	NA	D’Aubigne and Postel scores: 5.4 to 10.8	Stable bone ingrowth: 139 Stable fibrous ingrowth: 24 Unstable: 7	7 for mechanical failure	Greater than 95% at 13.2 years
Ng et al.	2004	Monoblock: AML and Solution	NA	23 (24)	5 (range, 2–10)	NA	NA	HHS: 93.1 (range, 80–100)	Stable bone ingrowth: 20 Stable fibrous ingrowth: 3 Unstable: 1	1 for deep infection	NA
Hamilton et al.	2007	Monoblock: AML, Solution, Prodigy and PFR.	NA	905	5.8 ± 5.5	NA	NA	NA	NA	12 for AL 4 for PJI 3 for stem fracture 1 for PFF	97.5% at 5 years and 95.9% at 10 years
Chung et al.	2012	Monoblock: AML and Solution	8 cases (8.3%)	96 (96)	5.5 (range, 2.0–11.3)	Type IIIA: 89 Type IIIB: 7	8 (100%)	HHS: 92.3 ± 8 (range, 77–100)	Stable bone ingrowth: 92 Stable fibrous ingrowth: 1	None	100% at 5.5 years
Ahmet et al.	2018	Monoblock: Echelon	13 cases (18.6%)	66 (70)	7.8 (range, 3.7–17.2)	Type I: 14 Type II: 27 Type IIIa: 29	NA	HHS: 72 (range, 43–96)	NA	AL: 1	98.4% at both 5 and 10 years
Emerson et al.	1992	NA	138 cases (100%)	107 (114)	2.1 (range, 0.5–5.5)	NA	133 (96.4%)	HHS: 79.6 (58 patients only)	Subsidence over 1 cm: 8	8 for mechanical failure	NA
Pak et al.	1993	Monoblock: AML	95 cases (100%)	95	4.75 (range, 2–8)	NA	88 (92.6%)	D’Aubigne and Postel scores: 4.2 to 8.7 (union)	NA	AL: 8	92.9% at 4.75 years
Head et al.	2000	NA	251 cases (100%)	251 cases	9.5 (range, 8–12)	NA	251 (100%)	HHS: average improvement of 45	NA	8 cases	NA
Barden et al.	2001	Monoblock: Solution	20 cases (100%)	20	4.7	Nonsupportive diaphysis	20 (100%)	HHS: 75.7 (range, 57.5–92)	Stable bone ingrowth: 17 Stable fibrous ingrowth: 3	3 mechanical failure	NA
Mokka et al.	2013	Extensively porous-coated stem: 31 Fluted distal fixation stems: 9	40 cases (100%)	40	4.3 (range, 1.0–10.4)	Type I: 5 Type II: 8 Type IIIA: 8 Type IIIB: 6 Type IV: 3	37 (92.5%)	NA	Stable: 36 Unstable: 4	4 for AL	NA
Kim et al.	2015	Monoblock: Solution	130 cases (100%)	120 (130)	16.1 (range, 12–20)	Type IIIB: 70 Type IV: 60	130 (100%)	HHS: 39 ± 10 to 86 ± 14 WOMAC: 62 ± 29 to 22 ± 19	Stable bone ingrowth: 113 Stable fibrous ingrowth: 5 Unstable: 12	10 for AL 2 for PJI	91% at 16 years
Current study	2019	Monoblock: Solution	31 cases (100%)	31 (31)	11.0 ± 1.5 (range, 8.1–13.5)	Type IIIA: 18 Type IIIB: 9 Type IV: 4	31 (100%)	HHS: 85.2 ± 6.6 WOMAC: 75.9 ± 10.4 SF-12: PCS 55.9 ± 18.3 MCS 60.0 ± 21.9	Stable bone ingrowth: 28 Stable fibrous ingrowth: 2 Unstable: 1	1 for AL	96.2% at 10 years

*NA* Not available

Although cortical strut allografts were previously considered unable to provide reliable direct support for the revision stems and were only used as bone augment for bone restoration, [31] several recent studies have demonstrated that cortical strut allografts can provide the primary prosthetic support for stems in nonsupportive diaphysis and achieve good clinical and radiographic outcomes [9, 11, 32]. In our study, initial axial and rotational stability of the stems could not be achieved without cortical strut allografts because of poor bone quantity and quality. The cortical strut allografts provided initial stability, reinforced the support for stems and augmented the bone stock. Cerclage wires were used to tighten the cortical strut allografts to the host bone and may also provide some support for the stems. The re-revision rate of the stems in our study was 3.2% after the application of allografts, and this rate was comparable to those presented in previous studies, although the extent of femoral bone loss was more severe in this study than in most prior studies [6, 9, 11]. The high survivorship of extensively porous-coated stems and the high union rate of cortical strut allografts suggest that this procedure represents a reliable approach to addressing severe femoral bone defects in revision THA.

In our series, we found that femoral width was significantly enhanced, indicating the reconstruction of femoral bone stock through the application of cortical strut allografts. When comparing femoral width between preoperative measurements and those obtained immediately postoperative and at the latest follow-up, mean increases of 10.5 mm and 7.8 mm, respectively, were observed. These results are in agreement with previously reported data [20, 33]. Poor femoral bone stock in revision THA has been shown to influence functional outcomes, [2] increase the risk of aseptic loosening, [5] and increase the risk of periprosthetic fracture [3] and also presents particular problems if further revision is required [4]; the restoration of bone stock is therefore of vital importance in eliminating the correlation between preoperative bone defects and poor clinical outcomes [12]. The majority of cortical strut allografts (74%) were graded as showing mild resorption, and others were graded as moderate in our series, comparable to the results reported in previous studies [11, 12, 31]. The slight decrease observed in femoral width (2.5 mm, 6% of immediate postoperative width) at the 10-year follow-up was consistent with the mild resorption of cortical strut allografts observed in this study. We speculate that the remodeling of cortical strut allografts progresses slowly after incorporation and that the improvement in bone stock was stable even at the 10-year follow-up.

The long-term durability of extensively porous-coated stems used with fibrous fixation after cortical strut allograft implantation remains a concern [9–12, 31]. Two stems in our study were assessed as stable fibrous ingrowth; one of these patients showed extremely thin cortical bone in the diaphysis in revision surgery and was assessed as having a Paprosky type IV bone defect, while the other patient suffered from progressive Alzheimer’s disease, and 6 years after revision surgery found it difficult to walk even with the assistance of crutches and had developed severe disuse osteoporosis. Although extensive radiolucent lines, no obvious endosteal spot welds and severe poor bone stock were found around the stems, no signs of stem subsidence were found at the latest follow-up. In addition, both patients reported acceptable hip function and no pain during ambulation or standing. We believe that both stems were stable and speculate that stems with fibrous fixation could be durable when supported by cortical strut allografts at a mean follow-up of 10 years. Our results are consistent with finding in previous reports indicating that fibrous fixation of the stem is correlated with osteoporotic femurs [34, 35].

A high incidence (range, 12–20%) of intraoperative fracture has been reported following procedures involving cementless long stems in revision THA with bone loss [36–38]. Only two intraoperative fractures (6.4%) occurred when the long stems were inserted in our series. Placing the cortical strut allografts prior to inserting the stems seems to be an effective way to provide extramedullary stabilization for the host bone and reduce the rate of intraoperative fracture. In addition, anterior bowing of the femur and thinning of the anterior cortex are common in Asian populations, and the use of stems with bows and smaller lengths consequently ensures that the maximum benefit is achieved in Asian patients [39]. In our series, 166-mm (6 in.) straight stems and 200-mm (8 in.) bowed stems were used in 13 and 18 patients in our study, respectively, thus ensuring the successful insertion of the long stems. Both fractures in our study were intraoperatively stabilized with cerclage wires and achieved union. No sign of stem loosening or re-fracture was observed at the ten-year follow-up. No fracture occurred when the previous stems were extracted in our series, and we propose that the application of ETO could minimize the risk of fracture when extracting well-fixed stems.

The present study has several limitations. First, the retrospective nature of this study led to inevitable bias in patient selection. However, it has been reported that in the majority of revision THA cases with Paprosky type III and IV femoral bone defects, the host bone was not able to provide reliable initial stability for extensively porous-coated stems and needed cortical strut allografts to provide additional stability [6, 9, 40]. In accordance with previous findings, more than 90% of the patients (39 out of 43) with Paprosky type III and IV defects at our institution needed cortical strut allografts to provide additional stability for the extensively porous-coated stems. We believe the patients included in this study offer a good representation of revision THA cases with type III and IV femoral bone defects. Second, 13% of the patients were lost to follow-up at the latest follow-up and were therefore excluded from this study. It should be noted that this also added to the selection bias of this study. However, we believe this rate may be acceptable for a minimum 8-year follow-up study. Third, the number of patients included in this study was relatively small. Further studies with larger sample sizes and longer follow-up periods are needed to confirm our findings.

## Conclusion

Our data suggest that extensively porous-coated stems combined with cortical strut allografts can provide satisfactory clinical and radiographic outcomes in patients who undergo revision THA with severe femoral bone defects after a minimum follow-up of 8 years. Further studies with larger sample sizes and longer follow-up periods are needed to confirm the efficacy of this technique in the management of severe femoral bone defects.

## Data Availability

Data used and analyzed in this study are available from the corresponding author on reasonable request.

## Abbreviations

PFF: Periprosthetic femoral fracture
AL: Aseptic loosening
PJI: Periprosthetic joint infection
HHS: Harris Hip Score
WOMAC: Western Ontario and McMaster Universities Osteoarthritis Index
PCS: Physical component summary
MCS: Mental component summary
